# Exploring PPAR Gamma and PPAR Alpha’s Regulation Role in Metabolism via Epigenetics Mechanism

**DOI:** 10.3390/biom14111445

**Published:** 2024-11-13

**Authors:** Małgorzata Małodobra-Mazur, Monika Ołdakowska, Tadeusz Dobosz

**Affiliations:** Department of Forensic Science, Division of Molecular Techniques, Wroclaw Medical University, Sklodowskiej-Curie 52, 51-367 Wroclaw, Poland; monika.oldakowska@umw.edu.pl (M.O.); tadeusz.dobosz@umw.edu.pl (T.D.)

**Keywords:** PPARA, PPARG, metabolic syndrome, obesity, DNA methylation, histone modifications

## Abstract

Peroxisome proliferator-activated receptors (PPARs) belong to a family of nuclear receptors. To date, three types of PPARs, namely PPARα, PPARδ, and PPARγ, have been identified, demonstrating co-expression across numerous tissues. PPARγ is primarily distributed in adipose tissue, the colon, the immune system, and the retina, while PPARα is predominantly expressed in metabolic tissues such as brown adipose tissue, the liver, and the kidneys. Both PPARγ and PPARα play crucial roles in various cellular processes. Recent data suggest that the PPAR family, among other mechanisms, might also be regulated by epigenetic mechanisms. Our recent studies, alongside numerous others, have highlighted the pivotal roles of DNA methylation and histone modifications in the regulation of PPARγ and PPARα, implicating them in the deterioration of metabolic disorders via epigenetic mechanisms. This still not fully understood mechanism of regulation in the nuclear receptors family has been summarized and described in the present paper. The present review summarizes the available data on PPARγ and PPARα regulation via epigenetic mechanisms, elucidating the link between the development of metabolic disorders and the dysregulation of PPARγ and PPARα resulting from these mechanisms.

## 1. Introduction

Peroxisome proliferator-activated receptors (PPARs) belong to the family of nuclear receptors classified as intranuclear receptors, acting as transcription factors when activated [1]. To date, three types of PPARs have been found to be co-expressed in numerous tissues, but with various distributions throughout the organism. The identified PPAR nuclear receptors, namely PPARα, PPARδ, and PPARγ, are similar in structure and function [2]. The most conservative domain across the three types of receptors is the DNA-binding domain (DBD), which contains two zinc-binding sites. The ligand-binding domain (LBD) is the largest domain and has four main features: (1) the dimerization interface, (2) ligand-binding pocket, (3) coregulator-binding surface, and (4) activation function 2 (AF2) [3].

In general, PPARs, after being activated by a specific ligand, bind to the RXR receptor to create a heterodimer and further regulate the expression of numerous genes. PPARγ ligands lead to the activation of insulin sensitization genes, mainly involved in adipogenesis, macrophage metabolism, and inflammatory genes [3,4]. The activation of PPARα leads to the upregulation of enzymes involved in fatty acid uptake, transport into mitochondria, and subsequent oxidation.

Insulin resistance, as well as type 2 diabetes, are classified as disorders in which an epigenetic component is strongly emphasized [5]. Epigenetics is defined as changes in gene function that are inherited by mitotic or meiotic cells and are not related to changes in the DNA sequence [6]. These changes might either enhance or reduce gene expression [7]. Epigenetics is linked with numerous diseases and disorders including cancer, neurodegenerative diseases, and metabolic disorders. Environmental factors have a significant impact on the DNA methylation profile and histone modifications, leading to the dysregulation of the expression of numerous genes, including insulin signaling and lipid metabolism genes [7]. Numerous studies have emphasized the impact of nutrition on human health, mainly via the epigenetic regulation of numerous processes required for maintaining homeostasis. The mechanism linking nutrition with epigenetic modifications is considered as a factor initiating or leading to numerous disorders, especially metabolic disorders [8].

Epidemiological studies consistently demonstrate a positive association between high-fat and carbohydrate-rich diets and the incidence of insulin resistance and type 2 diabetes. Furthermore, sedentary lifestyles exacerbate these effects, highlighting the intricate interplay between genetic predispositions and environmental influences in the pathogenesis of metabolic diseases [9,10]. Understanding the complex interplay between genetic predispositions and environmental factors is essential for elucidating the pathogenesis of metabolic diseases such as insulin resistance and type 2 diabetes.

The interplay between environmental factors and genetic predispositions is identified as one of the many factors contributing to the development of insulin resistance and type 2 diabetes mellitus (T2DM). Epigenetic modifications, such as DNA methylation and histone modifications, are increasingly recognized as crucial mediators in this relationship [7]. Numerous environmental conditions, as mentioned above, are known to induce epigenetic changes, thereby potentially predisposing individuals to metabolic disorders. Based on numerous studies, insulin resistance, obesity, and T2D have been shown to exhibit distinct alterations in the epigenome that result in the dysregulation of the key gene expression patterns involved in insulin signaling and/or lipid metabolism. However, the complexity of genetic and environmental interaction might also result from the inherited patterns of epigenetic changes, as these modifications can be transmitted via the placenta or sperm, influencing the offspring’s health and predisposing them to the development of metabolic disorders [11].

Epigenetic modifications are closely related to numerous diseases and disorders, including metabolic disorders. The present study describes and unifies available data regarding the importance of PPARγ and PPARα in proper insulin signaling and glucose and lipid metabolism via mechanisms connecting these nuclear receptors with epigenetic modifications. The present review describes the role of nuclear receptors in the pathogenesis and development of metabolic syndrome.

## 2. The Role of PPARγ and PPARα in Insulin Signaling and Glucose and Lipid Metabolism

*PPARG* is predominantly distributed in adipose tissue, the colon, the immune system, and the retina [12]. Four various mRNA transcript variants (*PPARG*1–4) are generated through alternative splicing. PPARγ plays numerous biological roles, including in the development, distribution and metabolism of adipose tissue [4]. PPARγ is the primary regulatory factor that controls the insulin signaling pathway and overall insulin sensitivity, and is necessary for the proper function of mature adipocytes [13,14,15]. Two main isoforms of PPARγ are distributed: PPARγ1 and PPARγ2, where the latter is mostly restricted to adipose tissue; however, the expression can be induced elsewhere by HFD [16]. It also plays an essential role in cell differentiation, and the regulation of apoptosis. Moreover, PPARγ inhibits inflammatory processes, exhibits anti-atherosclerosis activity, and improves heart performance [4,12,17]. First of all, PPARγ is the main agent that regulates adipogenesis by interaction with other genes that are necessary for the proper maturation of adipocytes (SRBP, FABP4). In terms of metabolic pathway regulation, the heterodimer PPARγ: RXR, and particularly PPARγ2, has been detected on the following target genes of the glucose metabolism pathway: *H6PD*, *PGD*, *GPI1*, *RPIA*, *PFKL*, *PTI1*, *GPD1*, *PDK1*, and *PCK1*. It also regulates lipid metabolism genes, including *GPAT3*, *LPN*, *LPL*, *CD36*, *ACSL1*, *LIPE*, *PNPLA2*, and others [17,18,19]. Additionally, it also has been shown to regulate the expression of the adiponectin gene (*ADIPOQ*), adiponectin receptor (*ADIPOR2*), and uncoupling protein 1 (*UCP-1*), and suppress the expression of inflammatory genes [20,21].

Numerous compounds act as PPARγ ligands, including both natural and synthetic substances. The natural agonists include docosahexaenoic acid, eicosatetraenoic acid, other polyunsaturated fatty acids, and some monounsaturated fatty acids. The most well-known group of synthetic ligands of PPARγ are the thiazolidinediones, such as troglitazone, rosiglitazone, and pioglitazone [22]. Numerous agonists of PPARγ have been shown to exhibit positive effects in type 2 diabetic patients, increasing insulin sensitivity, lowering blood glucose levels, and regulating lipid metabolism. Thus, several synthetic PPARγ ligands are successfully used for the treatment of metabolic disorders, including type 2 diabetes [2,4].

PPARα is distributed in numerous metabolically active tissues, mainly in the liver and tissues with an increased degree of mitochondrial oxidation and fatty acid catabolism, such as brown adipocytes, heart muscle, skeletal muscle, and the kidneys [12]. The role of PPARα in glucose homeostasis is not fully understood. PPARα plays a central role in regulating the expression of genes involved in fatty acid oxidation, lipid transport, and lipoprotein metabolism. It mainly promotes fatty acid utilization [12,23]. Through its effects on lipid metabolism, PPARα indirectly influences insulin sensitivity. The excessive accumulation of lipid intermediates, such as diacylglycerols and ceramides, in tissues like skeletal muscle and liver can impair insulin signaling, leading to insulin resistance [24]. Furthermore, it has been shown that PPARα agonists such as fenofibrate and Wy14643 can affect glucose homeostasis by increasing insulin sensitivity in adipocytes and muscle cells, which may be related to reduced lipid accumulation in cells through improved fatty acid β-oxidation [25,26]. It has been also suggested that PPARα impacts glucose homeostasis and indirectly affects pancreatic function. On the other hand, no relationship between fibrates and glucose homeostasis in humans has been demonstrated. Further research is needed to fully understand the role of PPARα in regulating blood glucose levels [26,27].

**Figure 1:**
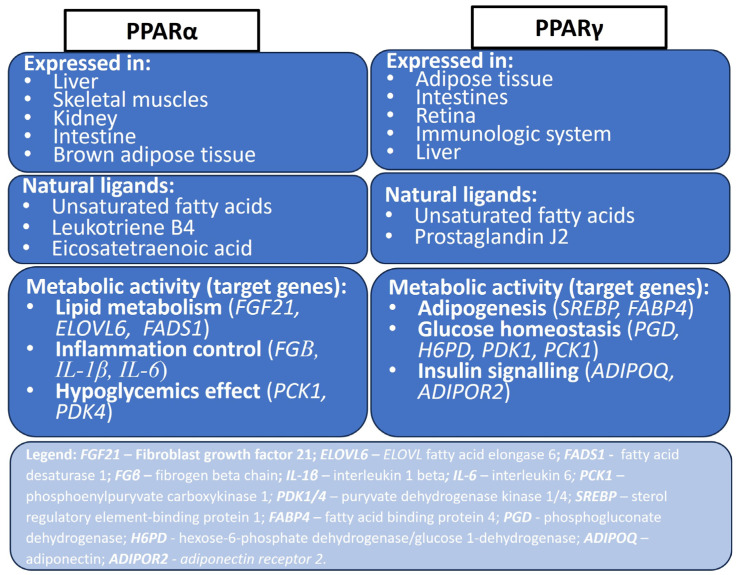
Main characteristic of two isoforms: PPARγ and PPARα. Their expression, function and the list of main ligands that activate the particular peroxisome proliferator are shown [18,36].

The significance of PPARα in lipid metabolism extends beyond its hepatic functions. In skeletal muscle, PPARα activation enhances fatty acid oxidation, providing an essential energy source during prolonged exercise or fasting states [28]. Moreover, in adipose tissue, it regulates adipocyte differentiation and lipid storage, impacting the overall energy balance [28,29]. PPARα plays a pivotal role in orchestrating the expression of genes involved in lipid uptake, oxidation, and synthesis [30]. The activation of PPARα leads to the upregulation of fatty acid oxidation enzymes such as acyl-CoA oxidase and carnitine palmitoyltransferase-1 [31], facilitating the breakdown of fatty acids for energy production [32]. Furthermore, PPARα is involved in the regulation of lipoprotein metabolism, particularly in the liver [33]. It enhances the expression of *ApoA-I* and *ApoA-II*, key components of high-density lipoprotein (HDL), contributing to the reverse cholesterol transport process. This function of PPARα aids in reducing the levels of low-density lipoprotein (LDL) cholesterol, thus playing a protective role against atherosclerosis [34]. The activation of this receptor also results in the induction of lipoprotein lipase (LPL), an enzyme crucial for the hydrolysis of triglycerides in circulating lipoproteins. Additionally, PPARα activation promotes the expression of APOC3, an inhibitor of LPL, thereby regulating the availability of free fatty acids for storage [35]. A summary of both PPARγ and PPARα expression and metabolic activity in various tissues of human body is presented in Figure 1.

## 3. The Relationship Between Nuclear Receptors and Epigenetic Mechanisms Driving Metabolic Diseases

The interaction between genetic predispositions and environmental influences plays a pivotal role in the pathogenesis of these disorders. Notably, dietary patterns rich in carbohydrates and fats, as well as processed foods, coupled with low physical activity, have been implicated in exacerbating the risk of developing insulin resistance and type 2 diabetes [37].

Peroxisome proliferator-activated receptors are crucial for proper cell metabolism, and any impact on the regulation of these genes substantially influences whole cell homeostasis and metabolism. It has been shown that *PPARG* is among the first genes divergently modified in newly onset insulin resistance [38]. In this context, exploring the complex interplay between epigenetic modifications and metabolic disorders holds promise for uncovering novel therapeutic targets and preventive strategies. A deeper understanding of these processes will enhance our ability to mitigate the burgeoning global burden of insulin resistance and T2D. Specific details of the collected data are presented below and summarized in Table 1.

### 3.1. Insights from DNA Methylation Studies

Various studies provide evidence for the simultaneous involvement of epigenetic and environmental factors in the development of metabolic diseases. The body of literature has demonstrated a clear relationship between alterations in DNA methylation and the histone modifications affecting various genes implicated in metabolic pathways. Considering the pivotal role of transcription factors in the regulation of the expression of numerous genes, significant attention has been paid to this context.

Numerous studies have indicated the pivotal role of DNA methylation in the regulation of the expression and proper function of *PPARG* in health and homeostasis, influencing many important life processes [17,19,38,39]. PPARγ is also considered to play a significant role in the pathogenesis of many diseases, particularly metabolic disorders and the epigenetic regulation of *PPARG*; in particular, DNA methylation has a significant impact, mainly by regulating *PPARG* expression. Consequently, the disruption of the expression of the *PPARG* gene can lead to various pathologies.

We have recently provided evidence that PPARγ undergoes epigenetic regulation, and any rearrangements lead to numerous metabolic disorders such as obesity or insulin resistance. Firstly, we have shown that the *PPARG* promoter is hypermethylated in obese and type 2 diabetic patients, which correlates with the downregulation of the expression of numerous genes responsible for proper insulin signal transduction in adipocytes [40]. In vivo studies have revealed that the *PPARG* promoter is hypermethylated in the adipose tissue of type 2 diabetic patients, both in visceral and subcutaneous adipose tissues. The hypermethylation positively correlated with the insulin resistance stage (assessed by HOMA-IR) and negatively with the expression of *PPARG*. Our observation has been supported by others who also demonstrated that epigenetic regulation has an impact on *PPARG* expression [41,42,43]. We and others have observed a distinct promoter methylation pattern in *PPARG* between various human fat depots, especially between subcutaneous and visceral adipose tissue. This observation likely arises from the fact that different fat depots (various types of adipose tissue) perform specific and distinct functions in the human body. We have observed considerable metabolic differences between SAT and VAT [44] concerning various aspects such as lipid metabolism, inflammatory state, insulin resistance induction, and lipid accumulation.

The observations gleaned from the in vivo investigation were subsequently replicated in vitro in a cell culture study, which enabled us to derive congruent conclusions. We showed the hypermethylation of the *PPARG* promoter, which correlated with the downregulation of *PPARG* expression in adipocytes with artificially induced insulin resistance [38]. Additionally, we demonstrated that in adipocytes with newly developed insulin resistance, global DNA methylation was increased, which correlated with the expression of *DNMT1* in those cells. Furthermore, the first gene to respond to changes in the DNA methylation profile due to high-fat diet-induced insulin resistance in adipocytes was *PPARG*. These changes in DNA methylation were observed as early as 72 h after insulin resistance induction by a palmitic acid (16:0), mimicking the high-fat diet. Our results might suggest that *PPARG*, acting as the transcription factor, may be the first response to the changing environmental conditions. No other analyzed genes showed dysregulation in either the expression rate or methylation profile after 72 h of insulin resistance induction [38].

The importance of epigenetic factors in the regulation of PPARs concerning metabolic diseases has been intensively studied by others as well. Volberg et al. observed differentially methylated CpG islands of the *PPARG* promoter in 9-year-old children; these negatively correlated with the birth weight and BMI of the children at the age of 9 years [43]. Another study found that a higher risk of type 2 diabetes is associated with the hypermethylation of the *PPARG* promoter in the pancreatic islets of diabetic patients, which negatively correlated with insulin secretion [45]. Similar results were obtained by Nilsson et al., where the hypermethylation of *PPARG* promoters was shown in type 2 diabetic patients compared to non-diabetes individuals in adipose tissue [46]. Epigenetic regulation has also been shown to impact *PPARG* regulation in non-human subjects. In overweight chickens, the promoter of *Pparg* was differentially methylated at three CpG positions compared to lean chickens [42]. A similar observation was made in db/db mice in terms of the hypermethylation of *Pparg* promoter in epididymal adipose tissue in comparison to wild-type animals; this negatively correlated with *PPARG* expression in the analyzed sample [47]. However, analyzing the above presented data, in vivo studies in both humans and animals should be carefully interpretated, as the analyzed tissues generally are composed of numerous various cells, where the expression profile of genes as well as their methylation status might vary.

The emerging role of epigenetic regulation, particularly DNA methylation, in *PPARG* function and action has been observed in various disorders. The dysregulation of *PPARG* methylation has been documented in idiopathic pulmonary fibrosis (IPF) patients. Wei et al. demonstrated the hypermethylation of *PPARG* in the lungs of IPF patients, which inversely correlated with the expression level and PPARG function [48]. Conversely, demethylation by 5′aza ameliorates the negative effect of IPF and restores the correct expression and function of *PPARG*. Similar results were obtained in the case of liver fibrosis, where the inflammatory state and liver fibrosis strongly correlated with the hypermethylation of *PPARG*, resulting in lower expression [49]. Furthermore, Hardy et al. proposed using the *PPARG* methylation status as a biomarker of liver fibrosis [50].

DNA methylation plays a crucial role in the regulation processes essential for adipogenesis, i.e., the formation of mature adipocytes. Proper epigenetic regulation is essential in this process, which, due to its specificity, is sensitive to external factors. Numerous transcription factors regulate adipogenesis, creating a network that can be easily disturbed [21]. PPARγ is a central regulator of adipogenesis because numerous genes possess PPARγ-binding sites. Thus, the hypermethylation of *PPARG* itself or its target genes might directly or indirectly link *PPARG* with the epigenetic regulation of adipogenesis and the metabolism of mature adipocytes, including a shift towards metabolic disorders. As PPARγ acts as the nuclear transcription factor, changes in the methylation profile of target genes might influence its binding to the specific response elements, thereby regulating adipogenesis. The importance of methylation-specific adipogenesis has been demonstrated, showing the significant role of PPARγ in the differentiation and function of mature adipocytes [21]. We have also previously demonstrated that the methylation of *PPARG* plays a crucial role in adipogenesis [51]. Moreover, we have shown that nutritional factors, especially fatty acids, play a significant role in methylome, including the methylation of *PPARG*. This impacts the differentiation process and the phenotype of mature adipocytes, shifting the adipocyte metabolism toward metabolic disorders [51,52]. There might be several possible mechanisms that affect how nutritional factors, especially fatty acids, influence DNA methylation [53]. First, fatty acids directly influence the expression and action of DNA methyltransferases. Second, ligands of various transcription factors might regulate epigenetic modification. Lastly, it has been proposed that fatty acids interact with MeCP2 (methyl CpG-binding protein (2), mainly in promoter regions regulating the expression of numerous genes [53].

Similar to PPARγ, its isoform PPARα also undergoes epigenetic regulation, including DNA methylation [54]. The promoter of *PPARA* is hypermethylated in type 2 diabetic patients with non-alcoholic fatty liver disease (NAFLD) [55]. Moreover, *PPARA* has been shown to undergo hydroxymethylation modifications that influence its expression, predisposing individuals to NAFLD and the development of metabolic syndrome [56]. Some studies have demonstrated the various DNA methylation patterns of *PPARA* in patients with metabolic syndrome and significant hyperlipidemia [57]. Castellano-Castillo et al. showed global hypermethylation in the visceral adipose tissue of patients with metabolic syndrome by assessing the methylation of LINE-1, which positively correlated with BMI and negatively correlated with insulin sensitivity (assessed by HOMA-IR index).

Furthermore, in addition to global DNA methylation, changes in the site-specific DNA methylation of numerous genes have been observed. These changes also correlated with metabolic dysregulation, including genes important for adipogenesis regulation, lipid metabolism, and inflammation. This suggests that DNA methylation, especially *PPARA*, *LPL*, *SCD* and *TNF-*α, is implicated in metabolism dysregulation and the pathogenesis of metabolic syndrome, involving adipose tissue metabolism dysregulation and the induction of the anti-inflammatory state [57].

### 3.2. Insights from the Histones Modifications Studies

Histone modifications are correlated with both the induction and downregulation of gene expression, depending on the site and type of modification. Generally, histone acetylation is associated with the induction of gene expression, while histone methylation, with some exceptions, is associated with gene expression downregulation. Histone acetylation maintains the negative charge of chromatic by removing the positive charge from the histone tail, neutralizing it, and thereby reducing its interaction with negatively charged DNA. As a result of chromatin relaxation, DNA becomes more accessible to numerous transcription factors [58]. Histone methylation can involve the mono-, di- or trimethylation of lysines or arginines of histone tails, and its effect on gene expression can be either enhancing or repressing, depending on the site and number of methyl groups added [59]. Our results clearly indicate the emerging role of histone modification in the induction of metabolic disorders, including obesity and insulin resistance. Notably, we have shown a global negative correlation between specific changes (H3K4me3 and H3K9/14ac) and insulin resistance, assessed by HOMA-IR [5]. Additionally, we have shown the downregulation of *SIRT1* and *SIRT7*, a key family of histones deacetylases in adipocytes with insulin resistance.

Lastly, we observed the lower enrichment level of H3K4me3 and H3K9/14ac within the *PPARG* promoter, which are the main markers of chromatin induction, corresponding with lower expression. Similar histone modifications and *SIRT7* downregulation were observed in both visceral and subcutaneously derived adipocytes, indicating a similar mechanism of epigenetic regulation in both fat depots. Furthermore, we previously demonstrated that *SIRT1* and *SIRT7* positively correlated with the expression of numerous genes involved in insulin signaling *(INSR*, *PIK3R1*, *AKT*, *SLC2A4*) and lipid metabolism (*ACC*, *FASN*, *SCD-1*, *LPL*), including *PPARG*. This suggests the emerging role of histone-modifying genes in the regulation of energy metabolism [5]. Indeed, numerous other researchers have shown the regulatory role and impact of Sirtuin family genes on the pathogenesis of metabolic disorders [60,61]. However, some data have indicated a negative correlation between *PPARG* and *SIRT1* [62].

Specific histone modifications have previously been correlated with divergent *PPARG* expression and the development of metabolic disorders. Castellano-Castillo et al. [63] reported the lower H3K4me3 enrichment of the *PPARG* promoter in adipose tissue from obese individuals compared to lean patients, suggesting an association with increased BMI and subsequent metabolic deterioration. Histone acetylation is correlated with chromatin induction and gene expression enhancement. Thus, histone acetylases (HAT) are believed to be key epigenetic players in adipogenesis and the regulation of energy metabolism [64]. According to Lefterova et al., the increased enrichment of H3K9ac marks was observed at *PPARG* binding sites during adipogenesis [65]. In our study, we have shown the lower H3K9/14ac enrichment of the *PPARG* promoter itself, as well as downstream targeted genes [5], in insulin-resistant adipocytes. Among others, Wang et al. [66] demonstrated that the downregulation of *PPARG* decreased the expression of *SLC1A5*, leading to a predisposition to obesity and insulin resistance. Additionally, *PPARG* expression was regulated by H3K27ac or H3K4me3.

**Table 1:** Epigenetic regulation of *PPARG* and *PPARA* and its effect on metabolism regulation, mainly in relation to glucose and lipid metabolism as well as insulin sensitivity.

Epigenetic Modification	Gene of Interest	Sample Type	Observed Effects	Pathway	References
Promoter hypermethylation	*PPARG*	Human adipose tissue SAT and VAT—in vivo study	Downregulation of *PPARG* expression as well as other insulin signaling and lipid metabolism genes. Insulin resistance and dysregulation of lipid levels	Insulin signaling Lipid metabolism	[40,46]
		Adipose tissue—animal model in vivo study	Metabolic syndrome development	Lipid metabolism	[47]
Promoter hypermethylation	*PPARG*	Human adipose tissue: surface and deep—in vivo study	Hypermethylation in deep adipose tissue correlated with lower PPARγ protein content in fat depot, lower adipogenicity properties and sensitivity to adipogenic agents	Adipogenesis	[41]
Various promoter methylation pattern	*PPARG*	Chicken adipose tissue—animal model in vivo study	Lower methylation in adipose tissue of fat chicken Age-related promoter methylation	Adipogenesis Lipid accumulation in adipose tissue	[42]
Promoter methylation pattern	*PPARG*	Human blood—in vivo study	Methylation status of some CpG was negatively correlated with birth weight and increased risk of obesity. Impact of birth weight on metabolism	Association with perinatal factors Hypothesis of programming metabolism	[43]
Promoter and gene body hypomethylation	*PPARG*	Human adipocytes—in vitro study	Hypomethylation of *PPARG* promoter during adipogenesis influences the rate of adipogenesis, lipid accumulation and phenotype of mature adipocytes. Hypomethylation is promoted by fatty acids supplementation	Adipogenesis	[21,51]
Promoter hypermethylation	*PPARA*	Hepatocytes—animal model in vitro study	Downregulation of mRNA and protein level of PPARα. Relationship with the pathogenesis of non-alcoholic fatty liver disease	Disruption in lipid accumulation	[55]
Global and gene-specific hypermethylation	*PPARA*	Human VAT—in vivo study	Dysregulation of gene expression including PPARα and downstream genes that strongly positively correlated with TG level	Metabolic syndrome development	[57]
Epigenetic changes related to circadian clock by H3K27ac and H3K4me3	*PPARG*	Adipose tissue—animal model in vivo study	Downregulation of PPARγ was a consequence of histone modification at H3K27ac or H3K4me3 leading to downregulation of further genes, including *SLC1A5*	Downregulation of SLC1A5 and further reduction in glutamine and methionine uptake	[66]
Changes at H3K4me3 and H3K9/14ac	*PPARG*	Human VAT and SAT—in vivo study	Dysregulation of *PPARG* and correlation with insulin resistance. H3K4me3 enrichment of *PPARG* directly correlated with BMI	Insulin signaling Lipids metabolism	[5,63]
Sirt-1-dependent action	*PPARG*	Adipocytes—in vivo and in vitro animal model study	Downregulation of PPARγ, lower mRNA and protein content	Lipolysis	[65]

The opposing epigenetic-modifying enzymes, specifically the HDAC family, have also been implicated in both adipogenesis and metabolic regulation via PPARs, mainly *PPARG* and *PPARA*. HDACs inhibitors have been shown to be involved in fatty acid metabolism and to play a protective role in the development of diabetic cardiomyopathy [67]. HDAC inhibitors also protect against atherosclerosis by enhancing the expression of *PPARG* [68]. The inhibition of HDACs maintains a higher rate of chromatic acetylation, which is a primary chromatin activation marker. According to Fu et al., the possible mechanism linking the activity of HDACs with the PPAR nuclear family (both *PPARG* and *PPARA)* is Cyclin D1 [69]. However, other mechanisms of interaction between *PPARG* and histone-modifying enzymes are possible and remain to be elucidated.

**Figure 2:**
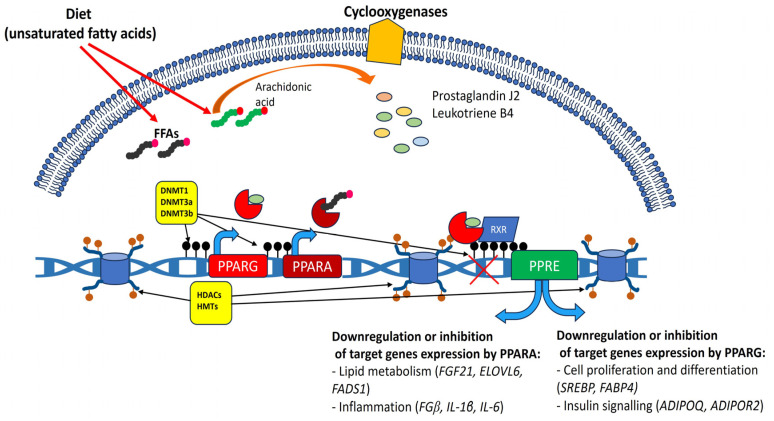
Schematic mechanism of epigenetic regulation involved in PPARγ and PPARγ expression and action, and their effects on cell metabolism. DNMT—DNA methyltransferases; HDAC—histone deacetylases; HMT—histone methylases, FGF21—Fibroblast growth factor 21; ELOVL6—ELOVL fatty acid elongase 6; FADS1—fatty acid desaturase 1; FGβ—fibrogen beta chain; IL-1β—interleukin 1 beta; IL-6—interleukin 6; SREBP—sterol regulatory element-binding protein 1; FABP4—fatty acid-binding protein 4; ADIPOQ—adiponectin; ADIPOR2—adiponectin receptor 2 (graph was prepared using PowerPoint software 2021).

The potential mechanism linking epigenetics (both DNA methylation and histone modification) with PPARs and the regulation of their expression is presented in Figure 2.

## 4. Conclusions

To conclude, the above data provide evidence that epigenetic regulation is one of the main mechanisms controlling PPAR action, which directly or indirectly affects the downstream genes responsible for metabolism regulation. Discrepancies in epigenetic regulation, such as dysregulation in the DNA methylation of histones modifications, might lead to disruptions in homeostasis and consequently contribute to the pathogenesis of metabolic disorders.

Understanding epigenetic regulation is crucial for the prevention, prediction, and future treatment of metabolic disorders. It is very likely that epigenetic-modifying agents could be effective in managing metabolic disorders.

## Data Availability

Not applicable.
